# Genomic Sequencing to Diagnose Prosthetic Joint Infection in the Knee: A Case Report

**DOI:** 10.7759/cureus.38788

**Published:** 2023-05-09

**Authors:** Hollie Wilkinson, Helen S McCarthy, Jade Perry, Tony Smith, Karina Wright, Paul Cool

**Affiliations:** 1 Bioengineering, Keele University, Keele, GBR; 2 Spinal Studies, The Robert Jones and Agnes Hunt Orthopaedic Hospital, Oswestry, GBR; 3 School of Pharmacy and Bioengineering, Keele University, Keele, GBR; 4 Surgery, The Robert Jones and Agnes Hunt Orthopaedic Hospital, Oswestry, GBR; 5 Orthopaedic Oncology, The Robert Jones and Agnes Hunt Orthopaedic Hospital, Oswestry, GBR; 6 Medical Sciences, Keele University, Keele, GBR

**Keywords:** orthopaedics, nanopore, diagnosis, infection, genomics

## Abstract

There is currently no "gold-standard" method to diagnose prosthetic joint infections (PJI), and the current practice of using microbiological cultures has many limitations. The identification of the bacterial species causing the infection is crucial to guide treatment; therefore, a robust method needs to be developed. Here, we attempt to use genomic sequencing with the MinION device from Oxford Nanopore Technologies to identify the species of bacteria causing PJI in a 61-year-old male. Genomic sequencing with the MinION presents an opportunity to produce species identification in real-time and at a smaller cost than current methods. By comparing results with standard hospital microbiological cultures, this study suggests that nanopore sequencing using the MinION could be a faster and more sensitive method to diagnose PJI than microbiological cultures.

## Introduction

Prosthetic joint infection (PJI) is commonly caused by the invasion of bacteria into a patient's wound during prosthetic joint implantation [1] and occurs at a rate of one to two percent depending on the type of surgery. Most surgical infections occur within the first year following surgery due to the invasion of microbes into a patient's wound before it is fully healed. Clinical features of PJI include pain, swelling, redness, and pyrexia. An infection can have serious consequences if not treated quickly [2] and correctly which can result in prolonged hospital stays and further surgeries. Once an infection is established in a patient, it is important to identify the bacteria that causes the infection so the correct antibiotics can be administered.

A faster and more accurate diagnosis of PJI can allow earlier intervention and improvements in patient outcomes [3]. There is currently no "gold standard" for diagnosing PJI, and there is no methodology routinely used for diagnosis and patient management [4]. Currently, clinicians use a combination of clinical features, blood test results, culture results, radiological imaging scans, and histopathological reviews to identify PJI. When an infection is suspected, samples of blood and tissue near the suspected infection site are taken to the laboratory for culturing of the causative microorganism. The results from these cultures are used to guide antibiotic treatment regimes. Microbiological cultures have limitations and have been reported to have low sensitivity in diagnosing infections relating to prosthetic implants. False-negative results are commonly reported, particularly if the patient has already been treated with some antibiotics or the bacterial load is not particularly high [4]. Contamination with subsequent false-positive results is also a reported problem with microbiological cultures [5]. The aim of this case report was to investigate if genomic sequencing could be a useful tool in identifying the bacterial species causing PJI. Furthermore, the abundance of the identified *Escherichia coli* species correlates with the severity of the infection.

Nanopore sequencing has led to the development of new sequencing devices such as the MinION device which was introduced in 2012 by Oxford Nanopore Technologies. This method of sequencing relies on electrical current changes caused by the passing of a negatively charged fragment of DNA through nanopores. The changes in the current through the nanopore created by the DNA fragment are then interpreted to produce a squiggle plot which is then translated into a nucleotide sequence. The MinION is a small portable device that allows single-molecule sequencing and can generate long reads in short time frames [6]. To use the MinION device, it is simply connected to a computer, and it is designed to be user-friendly. The MinION device allows the loading of single samples and fast output of the DNA sequences detected. The portability of the MinION device gives it the potential for sequencing at the site of sample collection, eliminating the need for transportation of the sample. The MinION is fairly straightforward to use; thus, it should be suitable for clinical use as there is no need for extensive training. The sequencing process is much quicker than other devices and should be possible in well under one and a half hours as the raw sequencing data is available to the user immediately. This device is relatively cheap compared to other sequencing technologies, making it attractive for clinical implementation as it only costs around 100 pounds per patient to sequence. The small and portable design means the MinION device has been used in field work for investigating disease outbreaks such as the Ebola virus, and it has also been applied to investigate microorganisms in the biomass industry [6].

## Case presentation

A 61-year-old man who underwent a left total knee replacement 13 years ago presented with severe swelling and pain in the left knee. Blood biochemistry results showed an erythrocyte sedimentation rate (ESR) of 39 (mm/hr), c-reactive protein (CRP) of 174 (mg/L), WBC count of 13.4 (10*9/L), and neutrophil count of 11.9 (10*9/L), which are all above the normal range. A diagnosis of PJI was made, and debridement of the knee with retention of the implant was performed in an attempt to remove the infected tissue and control the infection. During the procedure, samples of prosthetic fluid were taken for microbiology cultures, and three samples were cultured over a seven-day period. All three cultures grew *Escherichia coli* within a 48-hour period and one grew *Paenibacillus*. As it only grew on one culture, *Paenibacillus* was deemed to be a contaminant. *Escherichia coli* was named as the causative organism, and intravenous Tazocin antibiotic treatment was commenced. Despite early intervention with debridement and aggressive intravenous antibiotic treatment, the five-day post-operative blood biochemistry results indicated the persistence of the infection with an ESR of 39 (mm/hr), CRP 174 (mg/L), and WBC 12 (*109/L). At seven days post-op, the patient’s knee remained swollen and uncomfortable. Blood tests showed ongoing elevation of the inflammatory markers (ESR of 104 (mm/hr), CRP of 150 (mg/lL), and WBC of 7.6 (10*9/L)), necessitating further surgical intervention.

A second debridement was performed where blood and prosthetic fluid specimens were taken for microbiological cultures and nanopore sequencing of the DNA. The DNA was extracted from the blood and prosthetic fluid samples using the MagAttract [7] kit from Qiagen in preparation for sequencing using the MinION [8]. The sequencing time was set to 15 minutes based on recommendations from the literature and indications from preliminary investigations [9]. Genomic sequencing data with the MinION was analyzed using the Basic Local Alignment Search Tool (BLAST) to classify the genomic sequences, as this is the current gold standard [10].

The microbiological cultures from this debridement showed no growth after 7 days of incubation. Genomic sequencing detected *Escherichia coli*, *Klebsiella pneumonia*, *Acinetobacter baumannii*, and *Pseudomonas aeruginosa* in both blood and fluid samples following classification with BLAST and further filtering in R 4.2.2 (R Core Team, R Foundation for statistical computing, Vienna, Austria) and the standard R base package (Tables 1, 2).

**Table 1:** The breakdown and approximate time taken for each step of using genomic sequencing to identify the species of bacteria present in a blood sample.

Step	Time (minutes)
DNA extraction (MagAttract kit from Qiagen)	60
Sample preparation	15
Sequencing (using the MinION)	15
BLAST (intel I9 and 16CPU)	14
Filtering (including Porechop, NanoFilt, and quality filters in R Studio 4.2.2)	10
Total	104

**Table 2:** The remaining species from classifying the sequence reads in the patient blood sample using the BLAST bacteria database and further filtering in R 4.2.2

Bacteria Species	Count	Quality score	Gap mean	Mismatch mean
Acinetobacter baumannii	280350	84.19	5.34	27.16
Escherichia coli	119251	83.77	6.61	32.11
Klebsiella pneumoniae	16981	84.43	5.64	25.30
Pseudomonas aeruginosa	71807	84.12	7.22	33.19

Classification in BLAST (outfmt 6) required a sequence to have 90% similarity with the reference genome for it to be classified as a match. The computer used had an Intel 19 processor and Ubunto version 20 with MinKNOW version 22.08.4 installed. BLAST was run using a Linux command line and powered by 16 cores to improve classification speed and only took around 796 seconds. The command line was used to specify the FastQ file from the sequencing run and then to analyze it using the bacterial database from BLAST (taxid 2) to classify the genomic sequences present and discard the human genomic sequences. The BLAST output for every sample was filtered using R 4.2.2, where the classified genomic sequence required a quality score of more than 80 to be included in the final output as this would eliminate low-quality sequences that may have been incorrectly classified. A classification result was also required to have an average mismatch mean of less than 50 for the sequences detected to eliminate sequences that have a high number of bases that do not match the reference sequence. A result was also required to have a count of greater than 10,000 to eliminate those with a very low abundance in the sample. Finally, the species list was filtered to only include species previously associated with PJI. Therefore, any bacteria commonly found in the human flora are excluded from the results. Filtering using a predetermined list of species also helps rule out bacteria that are a contaminant, and these may be picked up from the sample collection or preparation process. Table 3 shows the breakdown of the steps involved in using genomic sequencing to identify the species of bacteria present in the sample and the approximate time for each step for this sample.

**Table 3:** The remaining species from classifying the sequence reads from the first fluid sample using the BLAST bacteria database and further filtering in R 4.2.2

Bacteria Species	Count	Quality mean	Gap mean	Mismatch mean
Acinetobacter baumannii	193403	84.49	4.91	25.18
Escherichia coli	81951	84.23	6.09	29.68
Klebsiella pneumoniae	11520	84.78	4.69	22.75
Pseudomonas aeruginosa	49891	84.37	6.12	30.26

Prior antibiotic treatment is identified in the literature as being a common cause of false-negative microbiological cultures grown to diagnose PJI, and this is a strong possibility in this patient [5]. The high count of *Escherichia coli* bacteria in the blood and fluid samples detected by genomic sequencing suggests that the antibiotic treatment did not clear the infection and it was still ongoing. The *Escherichia coli* count was significantly greater than that of *Klebsiella pneumoniae* and *Pseudomonas aeruginosa. *Therefore, it is likely that *Escherichia coli* is the more likely cause of the patient’s symptoms. *Escherichia coli* is a gram-negative, anaerobic bacterium often associated with clinical infections [11]. Some strains of *Escherichia coli* can cause serious illness in humans, such as urinary tract infections, gastroenteritis, and sepsis [12]. The detection of *Escherichia coli* should be recognized as important in a patient presenting with symptoms of PJI. *Escherichia coli* is highly virulent and more commonly associated with PJI than the other species identified by genomic sequencing in this case [13].

**Figure 1:**
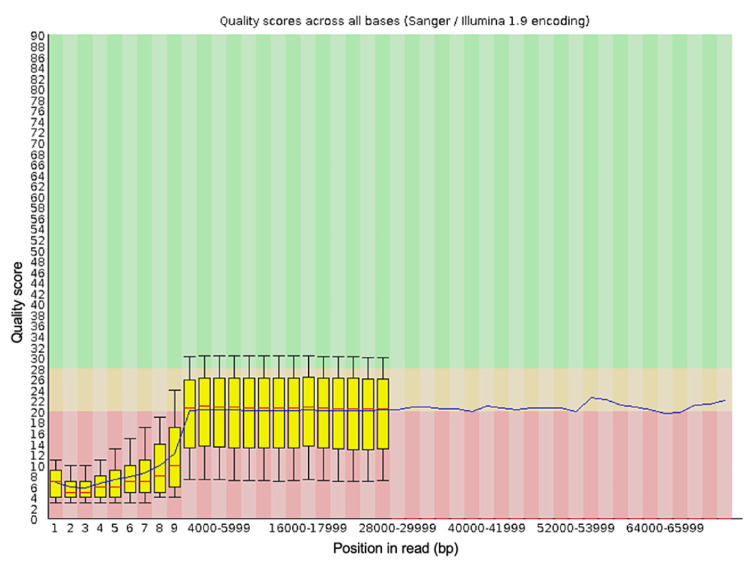
The per-base sequence quality for all of the reads generated from the blood sample from the first debridement procedure

**Figure 2:**
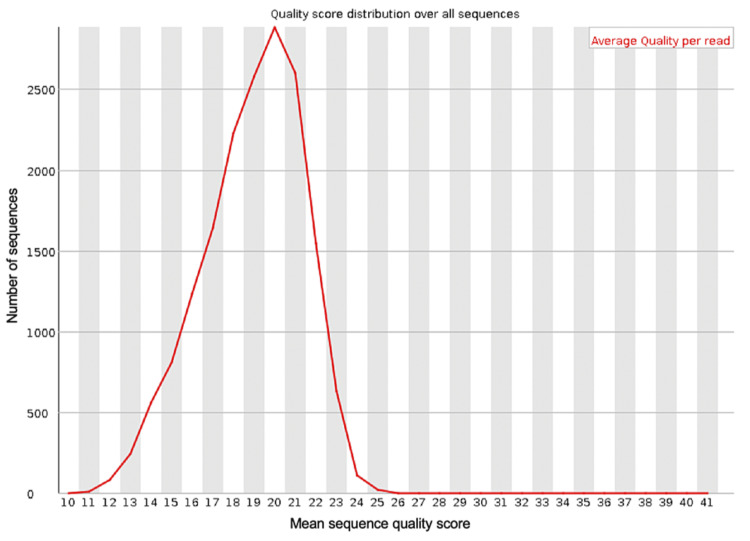
The mean PHRED quality scores (x-axis) for the genomic sequences generated from sequencing the blood sample from the first debridement procedure

We were able to perform extensive quality checks on the genomic sequencing data generated from the MinKNOW program from Oxford Nanopore Technologies. Extensive quality checks can be performed on the genomic sequencing data which improves the reliability of the results. When a sequencing run is complete, a number of files are generated containing information about the run, including summary statistics about the quantity and quality of the data produced. Sequencing the blood sample taken from the first debridement procedure generated 61.31 mb of data with approximately 19.36 k reads generated and no errors or warnings recognized. Zero sequences were flagged as being poor quality or identified as being overrepresented in the data. Using the command line, a number of other quality statistics were generated, including sequence quality scores (Figure 1), PHRED scores (Figure 2), and per-base quality scores.

The spread of quality scores for the sequences generated ranged from approximately 9.5 to 13, indicating some sequences were of poor quality. Figure 1 shows the per-base sequence quality for all of the reads generated in the run. This shows the mean and standard deviation of the sequencing quality at each position for all of the reads generated. This figure demonstrates that some of the reads at the beginning of the sequences were of much lower quality than at the base positions further along the sequences. The low-quality scores generated by some of the reads generated suggests that it may be necessary to trim the dataset to improve the overall quality of the data and, therefore, the reliability of the results after classifying the data using BLAST. Trimming of the dataset can be done using the Porechop (v0.2.4) and NanoFilt (v2.8.0) tools and can be applied to the FastQ files generated from the sequencing run using the command line. Adding quality control using these tools will not add any significant time to the data analysis. If trimming the FastQ file using these tools improves the quality scores, then the sequences should be reclassified using BLAST using the trimmed FastQ file.

**Figure 3:**
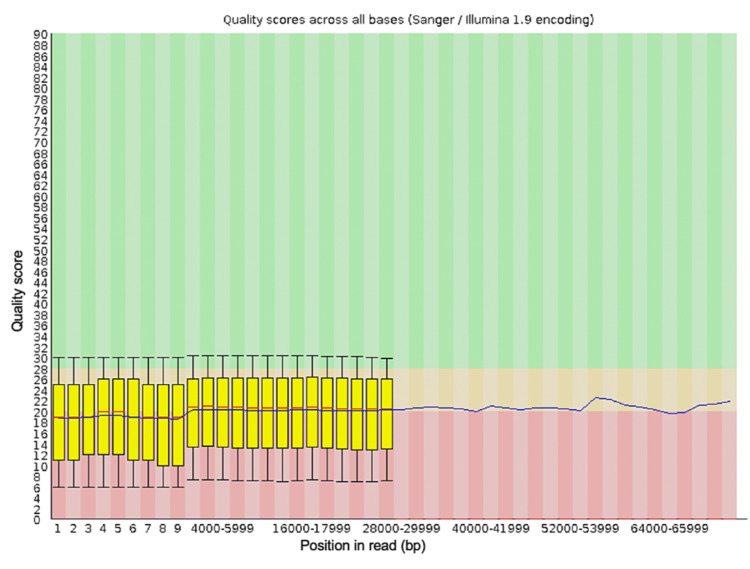
The per-base sequence quality for all of the reads generated from the blood sample from the first debridement procedure after trimming the data with Porechop and NanoFilt

**Figure 4:**
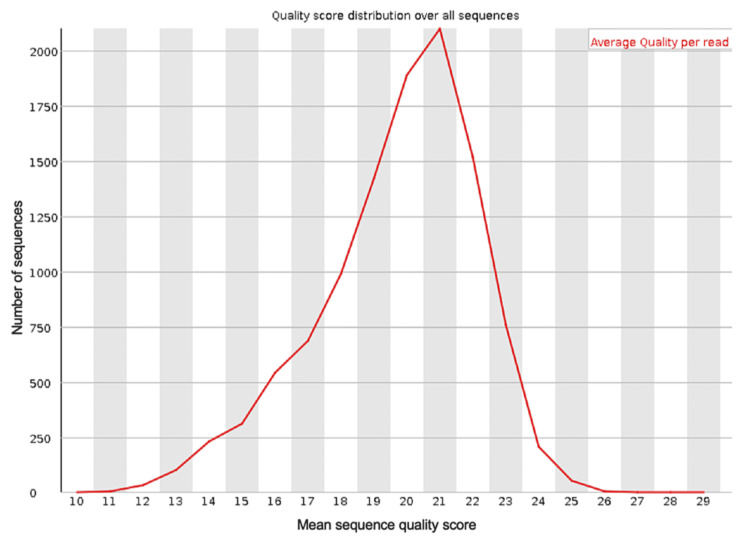
The mean PHRED quality scores (x-axis) for the genomic sequences generated from sequencing the blood sample from the first debridement procedure after trimming the data with Porechop and NanoFilt

Porechop is a tool created to trim adapter sequences from data generated by genomic sequencing with Oxford Nanopore Technologies. This tool will remove adapter sequences identified in the input FastQ file specified and then create a new FastQ file without these sequences, then analysis of the quality of the sequences in this new file can be done and compared to the original FastQ file. NanoFilt is a tool designed to trim specified short read sequences from a specified FastQ file. The output FastQ file from the first debridement was trimmed with Porechop to remove any adapter sequences, and then with NanoFilt to remove any sequences shorter than 500 nucleotides and the first 10 bases from each sequence. Trimming of the FastQ file using Porechop and Nanofilt can be done very quickly by running a single line of command for each once the required packages have been installed and take seconds. These tools can be included in the script to run BLAST analysis in the future. Running these tools creates a new FastQ file, and the quality of this file was analyzed to compare to the quality of the original FastQ file. The new quality scores for the trimmed FastQ file were better (Figures 3, 4) than the original FastQ file, these quality readings were generated using FASTQC.

Analysis with BLAST of the trimmed FastQ file identified the same bacterial species as the first FastQ file but with higher quality scores (Table 4).

**Table 4:** The count, quality mean, gap mean, and mismatch mean of bacteria species detected in samples from the first sample following trimming of the FastQ file using Porechop

Bacterial species	Count	Quality mean	Gap mean	Mismatch mean
Acinetobacter baumannii	283530	84.25	5.30	26.96
Escherichia coli	122918	84.10	6.45	31.26
Klebsiella pneumoniae	17568	84.77	5.52	24.63
Pseudomonas aeruginosa	73287	84.31	7.11	32.63

As the patient was not clinically improving, and swelling continued. Antibiotic treatment was changed to include intravenous ciprofloxacin. Several days later, the patient’s inflammatory markers improved (ESR of 77 (mm/hr) and CRP of 42 (mg/L)) but were still notably above the normal range. The knee remained swollen and very uncomfortable; thus, the removal of the implant was planned (two-stage revision).

In the following procedure, prosthetic fluid samples were taken for microbiological cultures and genomic sequencing. The microbiological cultures yielded no growth after incubation for seven days. The genomic sequencing data from the second debridement did not detect enough hits from *Escherichia coli* for it to be included in the output following filtering in RStudio. However, when searching for *Escherichia coli* in the classification output from BLAST, it was present, albeit at just below the threshold count for filtering (9171) (Table 5). The reduced count of *Escherichia coli* coincides with the reduced inflammatory markers (ESR of 77 (mm/hr) and CRP of 42 (mg/L)), suggesting the infection was subsiding. Following surgery, intravenous antibiotic treatment was continued for two weeks. Subsequently, the ESR settled to 11 (mm/hr), and the patient was discharged on a course of oral antibiotics.

**Table 5:** The count, quality mean, gap mean and mismatch mean of Escherichia coli detected in samples from the second fluid sample using genomic sequencing.

Bacteria species	Count	Quality mean	Gap mean	Mismatch mean
Escherichia coli	9171	91.02	3.74	15.23

**Figure 5:**
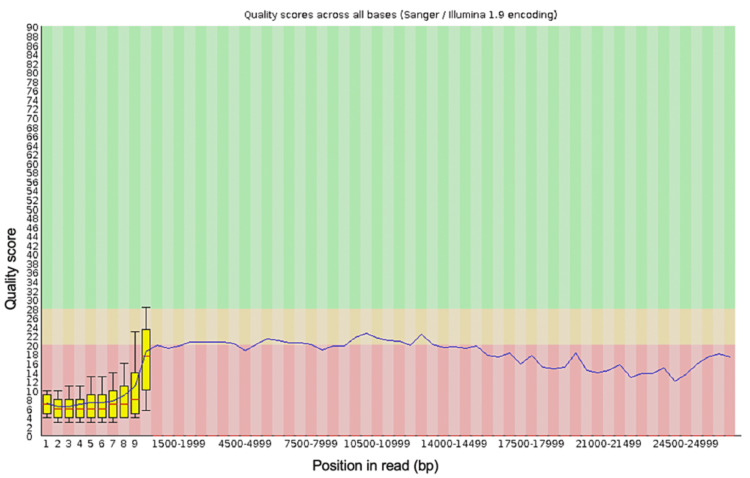
The per-base sequence quality scores for the sequencing data from the sample from the second debridement procedure

Quality checks were performed on the genomic sequencing data generated from the sample taken from the second debridement procedure. This sequencing run produced 448.62 kb of data with approximately 181 reads. No sequences from this run were tagged as being of poor quality, but the per-base sequence quality scores for some reads were very low from this sequencing run (Figure 5).

**Figure 6:**
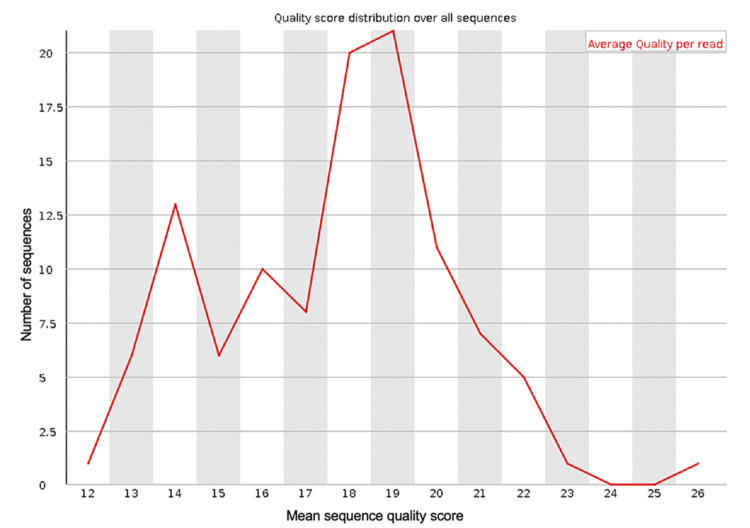
The mean PHRED quality scores (x-axis) of the genomic sequences identified in the sample from the second debridement procedure

There was variation in the quality scores for the sequences identified from this sequencing run (Figure 6). The low-quality score for some sequences suggests the data needs to be trimmed using the Porechop and NanoFilt tools to improve the quality of the data before classification using BLAST. The analysis identified several "overrepresented" sequences in this data set suggesting they need to be removed. This is also done by trimming the FastQ files. These sequences are often sequencing adapters, etc., and should be removed before the data is analyzed using BLAST. This was subsequently done using Porechop and NanoFilt tools in the command line.

**Figure 7:**
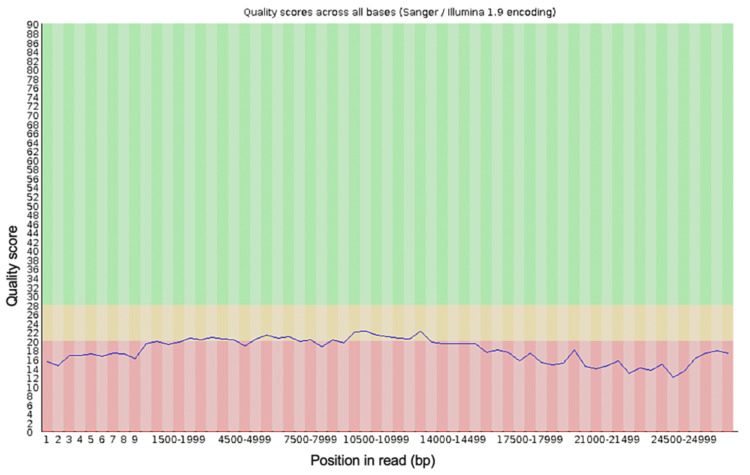
The per-base sequence quality scores for the sequencing data from the sample from the second debridement procedure after trimming the data with Porechop and NanoFilt

**Figure 8:**
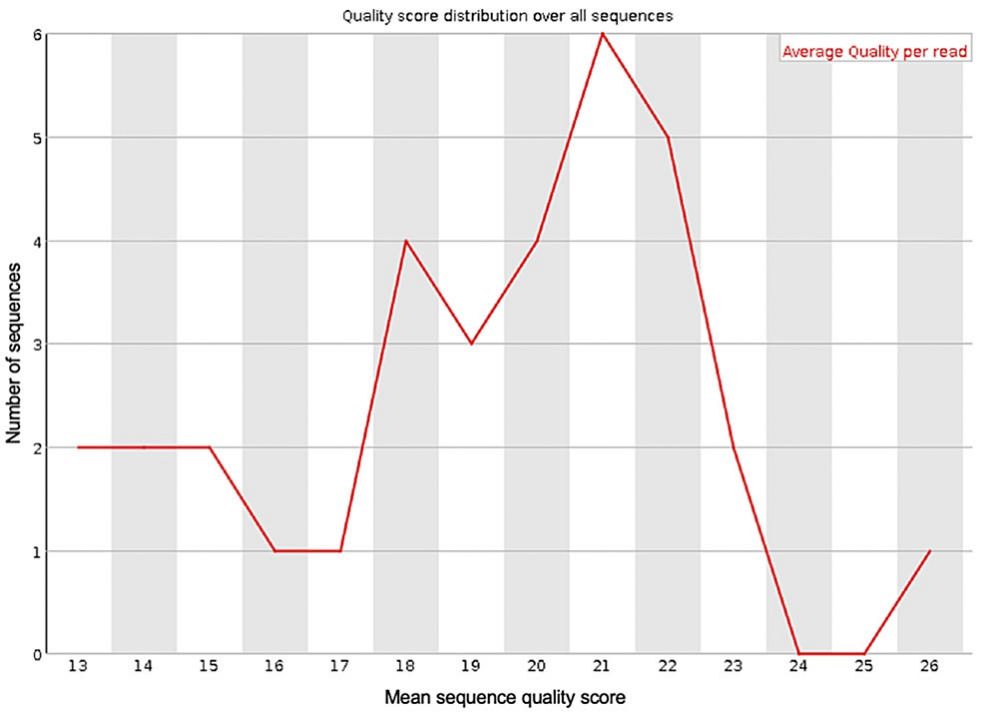
The PHRED quality scores (x-axis) of the genomic sequences identified in the sample from the second debridement procedure after trimming the data with Porechop and NanoFilt

The output FastQ file from the first debridement was trimmed with Porechop and then NanoFilt to remove any sequences shorter than 500 nucleotides and remove the first 10 bases of each sequence. This then creates a new FastQ file, and the quality of this file was analyzed to compare to the quality of the original FastQ file. The new quality scores for the trimmed FastQ file were better than the original FastQ file (Figures 7, 8).

## Discussion

The diagnosis of PJI is an ongoing problem with some of the biggest challenges including false-negative results. Currently, a combination of patient blood biochemistry, clinical presentation, and microbiological cultures are used, but there is no "gold standard" for diagnosing PJI. Patient inflammatory markers including CRP, ESR, and WBC are often raised in response to infection. However, they can also be raised in response to surgery and can remain raised for weeks, even when there is no ongoing infection. Blood and prosthetic fluid samples are frequently taken from patients who have received prosthetic joint replacement surgery and are suspected to have an infection. These samples are grown in culture to observe what species of bacteria grow and are causing the infection. There are many reasons that cultures may be negative despite there being an ongoing bacterial infection. These reasons include previous antibiotic treatment and poor sampling technique (Figure 1). Cultures also frequently produce false-positive results due to contamination of the sample from bacteria present in the normal human flora or during the culturing process. These contaminant bacteria often grow well on microbiological cultures which makes diagnosing PJI difficult. Although this can also be a challenge when producing genomic sequence data from these samples, there is the ability to filter the species list to exclude the bacteria we know that inhabits the normal human flora and are common contaminants of microbiological cultures [10]. The detection of other bacterial species is likely due to the lower read accuracy and reduced coverage of sequencing with the MinION [6].

Microbiological cultures are currently the most common method used to identify the bacterial species causing PJI. However, microbiological cultures frequently produce false-negative results particularly when the patient has previously been treated with antibiotics. Genetic sequencing techniques may be useful for detecting the presence of bacteria even if the concentration of microbes may be reduced following antibiotic treatment [3]. Fourth-generation nanopore sequencing can produce sequence data from single DNA molecules, suggesting that it may have a higher sensitivity than microbiological cultures for detecting the bacteria present in a sample [14]. The use of nanopore sequencing for this patient identified *Escherichia coli* when cultures yielded no growth, and the patient's clinical observations indicated the persistence of infection. Although sequencing detected bacterial species we may class as false-positive results (*Klebsiella pneumoniae* and *Pseudomonas aeruginosa*), they were detected at a significantly lower concentration than *Escherichia coli*, which was previously identified as the causative organism. In addition, when trying to diagnose PJI, it could be argued that false-positive results are less of an obstacle to making a diagnosis than false-negative results, which is a major drawback of using microbiological cultures. Despite the identification of bacterial species likely not causing the infection, genomic sequencing still identified the causative organism, and it presented as the second hit. These results can still be informative of the most appropriate antibiotics as it has correctly identified the causative pathogen when microbiological cultures have failed.

Weeks following the patient's discharge from the hospital and significant improvements in his condition, the patient returned to the hospital with significant swelling, redness, and pain in the knee. These symptoms coincide with the recurrence of the PJI in the knee. The return of these symptoms suggested that the suspected false-positive results from genomic sequencing may have been true positives. All or one of the other species detected (*Acinetobacter baumannii*, *Klebsiella pneumonia,* and *Pseudomonas aeruginosa*) may also be causative of the infection as well as *Escherichia coli*, as polymicrobial infections are not uncommon. The prescription of ceftriaxone antibiotics, which seemed to clear the established *Escherichia coli* infection, may not have been effective against the other bacteria species identified as antibiotics are generally prescribed based on the bacterial species identified. In this case, only *Escherichia coli* was identified from microbiological cultures. When the patient returned for the repeat revision surgery, specimens were again taken for microbiology culture, but there was no growth after seven days. Unfortunately, specimens were not taken for genomic sequencing as the repeat revision was an emergency procedure. Therefore, there was no opportunity to consent the patient.

The issue of false-positive findings may not always be an obstacle when diagnosing PJI. In many cases, different bacterial species can be treated with the same type of antibiotics. Thus, the detection of additional species may not impact the course of treatment. For example, beta-lactam antibiotics can be effective against a variety of bacterial pathogens commonly associated with PJI. The species that can be treated with beta-lactam antibiotics include *Staphylococcus aureus*, *Escherichia coli,* and *Klebsiella species*. These antibiotics work by targeting the peptidoglycan cross-links in the cell wall of bacteria, including some gram-positive and gram-negative species. These antibiotics can also be administered effectively orally and, thus, may be particularly useful where patients are going to be discharged [15].

By using genomic sequencing to look for bacteria present in the samples, *Escherichia coli* was identified as being present at a high concentration (count of 81,951 in the fluid sample and 119,251 in the blood) when microbiological cultures were negative, suggesting that *Escherichia coli* is still causing ongoing infection. When the genomic sequencing data was analyzed from the second debridement and following the change in antibiotic treatment, the final results did not show *Escherichia coli*. However, a search for *Escherichia coli* in the BLAST output showed it was present at a count of 9171, just only missing the cut-off of 10,000 applied when filtering. The quality mean and mismatch mean met the filter requirements in RStudio, suggesting that the *Escherichia coli* genome detected has high similarity to the reference sequence in the BLAST database (Table 3). It is important that some gaps and mismatches are allowed when classifying sequences with BLAST as they naturally occur through mutational events. Thus, there will never be a complete match with a reference genome.

Sequencing using the MinION from Oxford Nanopore has some identified limitations which may explain the detection of unexpected bacterial species [16]. There are limited reads generated per sequencing run from the flow cell which may result in reduced coverage and, therefore, classification accuracy of the genomic sequences produced. Sequencing and base calling using the MinION are very quick in comparison to other techniques, and this comes with a tradeoff of reduced quality reads. In addition, this technology produces relatively short reads in comparison with other sequencing techniques. This makes the filters applied to the data even more important as it is important in not eliminating too many results [17]. There is the possibility that the quantity of data produced from sequencing with the MinION could have impacted the classification results as there may not have been enough high-quality reads. Poor coverage of particular genomic sequences could lead to misclassification. This risk is likely increased by shortening the sequencing time, which was only 15 minutes in this case report [18]. Another potential drawback of the MinION is the GC sequencing bias. Genomic sequences with a lower quantity of GC reads have a lower error rate than those with a higher quantity of GC reads when sequenced with nanopore sequencing technology. The GC quantity can vary quite drastically between the genomes of different bacterial species; for example, *Acinetobacter baumannii* has a GC quantity of around 39%, whereas *Escherichia coli* has a GC quantity of over 50% [19]. This difference in GC quantity could have resulted in a bias in high-quality sequencing reads toward *Acinetobacter baumannii*, resulting in increased hits for this species and reduced hits for *Escherichia coli*. A greater quantity of GC bases in a genome could result in the genome sequence being present at a higher representation in the output sequence data. In addition, classification using BLAST can sometimes misclassify sequences. "Repetitive sequences violate certain assumptions made in the statistical theory that underlies BLAST. Sequence databases are known to include vector sequences and other sequencing errors" (Pertsemlidis and Fondon (2001)). BLAST results can include artifacts, such as adapters used in the methodology, and are recognized as not accurate in classifying sequences with repetitive regions [17]. Some species of bacteria have very similar genome sequences which can lead to misclassification, particularly when trying to classify to species level. For classification in this investigation, sequence similarity was set to 90% with the reference sequence in the BLAST database. This may lead to inaccuracies when identifying species that have high genomic sequence similarity and inevitably lead to false-positive classification results.

## Conclusions

In summary, this case report demonstrates how genomic sequencing can detect the presence of *Escherichia coli* in clinical samples from a patient with PJI when cultures did not. It indicated that genomic sequencing is likely a more sensitive method to use when attempting to identify the species of bacteria causing PJI as the current methods produce many false-negative results. The results produced by genomic sequencing can also undergo extensive quality checks which microbiological cultures cannot confirm the reliability of findings.

The identification of *Escherichia coli* using genomic sequencing in this case report suggests that genomic sequencing is useful in identifying bacterial species that cause PJI and should be considered as an alternative approach to the current gold standard of using microbiological cultures in the future.

## Figures and Tables

**Figure 9:**
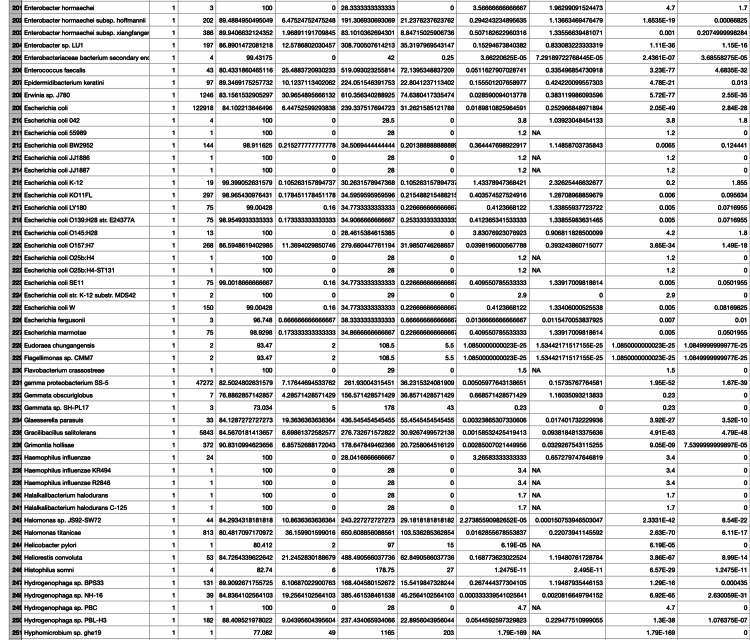
The BLAST output from running the genomic sequence data from the blood sample from the first debridement procedure

## Appendices

**Table 6:** The command line code for running PoreChop and Nanofilt to trim the FASTQ files produced from nanopore sequencing

PoreChop	https://github.com/rrwick/Porechop#quick-usage-examples
Nanofilt	https://github.com/wdecoster/nanofilt
